# Clinical feasibility of CS-VIBE accelerates MRI techniques in diagnosing intracranial metastasis

**DOI:** 10.1038/s41598-023-37148-3

**Published:** 2023-06-20

**Authors:** Sang Ik Park, Younghee Yim, Mi Sun Chung

**Affiliations:** 1 Department of Radiology, Chung-Ang University Hospital, Chung-Ang University College of Medicine, 102 Heukseok-ro, Dongjak-gu, Seoul, Republic of Korea; 2 Department of Radiology, Human Medical Imaging and Intervention Center, Seoul, Korea

**Keywords:** Medical research, Oncology

## Abstract

Our objective was to evaluate and compare the diagnostic performance of post-contrast 3D compressed-sensing volume-interpolated breath-hold examination (CS-VIBE) and 3D T1 magnetization-prepared rapid-acquisition gradient-echo (MPRAGE) in detecting intracranial metastasis. Additionally, we analyzed and compared the image quality between the two. We enrolled 164 cancer patients who underwent contrast-enhanced brain MRI. Two neuroradiologists independently reviewed all the images. The signal-to-noise ratio (SNR), contrast-to noise ratio (CNR) were compared between two sequences. For patients with intracranial metastasis, we measured enhancement degree and CNR_lesion/parenchyma_ of the lesion. The overall image quality, motion artifact, gray-white matter discrimination and enhancing lesion conspicuity were analyzed. Both MPRAGE and CS-VIBE showed similar performance in diagnosing intracranial metastasis. Overall image quality of CS-VIBE was better with less motion artifact; however conventional MPRAGE was superior in enhancing lesion conspicuity. Overall, the SNR and CNR of conventional MPRAGE were higher than those of CS-VIBE. For 30 enhancing intracranial metastatic lesions, MPRAGE showed a lower CNR (*p* = 0.02) and contrast ratio (*p* = 0.03). MPRAGE and CS-VIBE were preferred in 11.6 and 13.4% of cases, respectively. In comparison with conventional MPRAGE, CS-VIBE achieved comparable image quality and visualization, with the scan time being half of that of MPRAGE.

## Introduction

The timely detection and diagnosis of brain metastasis and accurate differentiation from other neuro-pathologies are crucial for cancer patients as it might affect the disease prognosis and treatment outcome. Although biopsy is often needed for a definite diagnosis, imaging diagnosis is essential to decide the treatment strategy, which can include systemic chemotherapy, stereotactic radiosurgery (SRS), and whole brain radiation therapy (WBRT)^1^. As current MRI provide good tissue contrast and great performance to modify various sequence to characterize brain lesions, contrast-enhanced MRI has been used as the modality of choice for assessing brain metastasis^2,3^. A clinically dedicated brain metastasis MRI protocol typically includes pre- and post-contrast sequences. Although there is still a debate regarding which post-contrast T1-wieghted imaging sequence is the best^4^, the post-contrast 3D T1-weighted sequence, which is a high-resolution sequence acquired by either 3D volumetric Fast Spoiled Gradient-Echo (FSPGR) or Fast Spin-Echo (FSE) technique are widely used in clinical field^1^. Currently, the MR protocol for brain metastasis consists of several advanced MR techniques such as proton MRS, diffusion or perfusion as well as conventional sequences to characterize and support the differentiation of metastases from other disease entities^4^. Consequently, the total scan time could be prolonged, which might result in motion artifacts^5^ and patient anxiety^6^.

Recently, compressed sensing (CS) has been applied to decrease the scan time without significant compromise of image quality by achieving higher acceleration through sparsity in the spatial domain^7^. For fast 3D T1-weighted imaging of the brain, the conventional Volume-Interpolated Breath-hold Examination (VIBE) was already reported to provide better signal-to-noise ratio (SNR) and contrast-to-noise ratio (CNR) compared with a magnetization-prepared rapid-acquisition gradient-echo (MPRAGE) sequence^8,9^. Moreover, a prototypical compressed sensing volume-interpolated breath-hold examination (CS-VIBE) sequence had been reported for dynamic liver and breast imaging^10,11^. However, CS-VIBE has not been applied and validated in brain imaging to evaluate brain lesions.

We hypothesize that similar diagnostic performance with acceptable image quality and a significantly shorter scan time may be achieved with CS-VIBE compared with conventional 3D T1 MPRAGE. In this study, we compared the diagnostic performance of post-contrast 3D CS-VIBE and 3D T1 MPRAGE in diagnosing metastasis. We also examined and compared the image quality of the two sequences.

## Materials and methods

We compare the diagnostic performance of conventional MPRAGE and CS-VIBE for the detection of brain metastasis and the image quality of both sequences. The methods and results have been reported in accordance with the Standards for Reporting of Diagnostic Accuracy Studies (STARD) guidelines^12^. All methods were approved by the Chung-Ang University Hospital Institutional Review Board (IRB) No. 2112-026-19397). The requirement for informed consent was waived for this retrospective study and was approved by Chung-Ang University Hospital, Institutional review board (IRB).

### Study population

**Figure 1:**
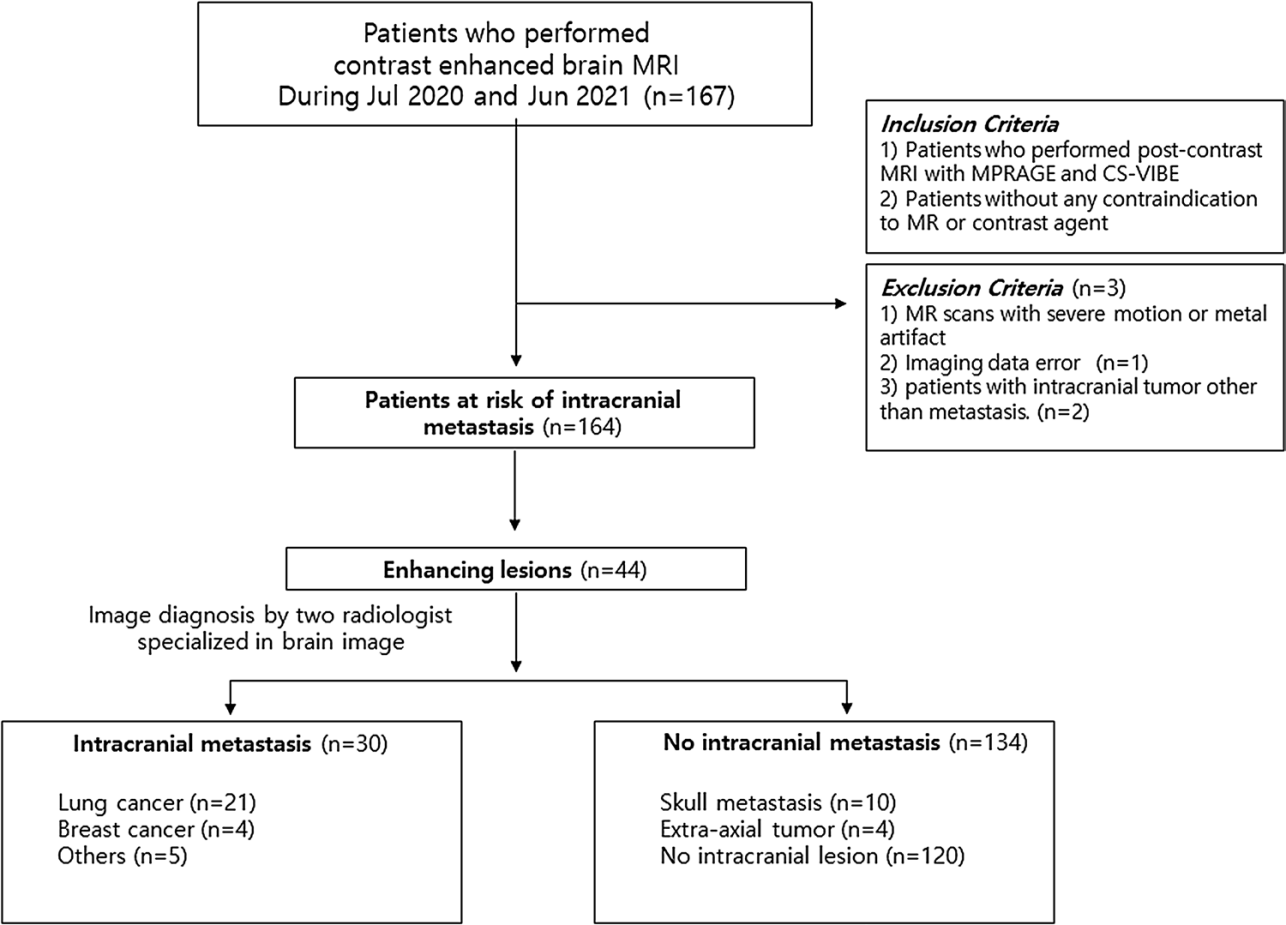
Study flow chart.

We retrospectively included 167 consecutive patients who had undergone contrast-enhanced cranial neve MRI from July 2020 to July 2021 in a single referral center (Fig. 1). The inclusion criteria were as follows: (1) patients who underwent contrast-enhanced brain MRI with conventional 3D T1 MPRAGE and 3D CS-VIBE to evaluate possible brain metastasis, and (2) patients without any contraindication to MR or contrast enhancement. The exclusion criteria were as follows: (1) MR scans with severe motion or metal artifact; (2) imaging data error; (3) patients with brain tumors other than metastasis. Three patients were excluded due to imaging reconstruction error (n = 1), and other brain tumors (n = 2, glioblastoma).

We retrospectively surveyed the demographic and clinical data by reviewing the electronic medical records, including age, sex and final diagnosis (Table 1).

**Table 1:** Study population.

	(n = 164)
Age, mean ± SD (range), years	66.5 ± 11.9 (33–99)
Male (n/total), %	55.5 (91/164)
Disease	
Lung cancer (n/total), %	66.5 (109/164)
Breast cancer (n/total), %	7.9 (13/164)
Others (n/total), %	25.6 (42/164)
Metastasis (n/total), %	18.3 (30/164)

Data in parentheses represent percentages or data ranges.

### Image acquisition

All MRIs were performed on two 3 T scanners (MAGNETOM Skyra, Siemens Healthcare, Erlangen, Germany) with 64-channel head coils. The detailed MR acquisition parameters of conventional and CS-VIBE are shown in detail in Table 2. Routine brain MRI with contrast enhancement was performed for 3D T1 MPRAGE and CS-VIBE sequences (prototype sequence provided by Siemens Healthcare). For CS-VIBE, image reconstruction was performed by an iterative optimization using the Fast Iterative Shrinkage-Thresholding Algorithm (FISTA)^13^ that was integrated into the reconstruction pipeline of the scanner. The cost function of the optimization comprised a SENSE-based data consistency term and the l1-norm of the Haar wavelet transform of the reconstructed image^14^ similar to the implementation reported previously.

**Table 2:** Image parameters.

	Conventional MPRAGE	CS-VIBE
Field of view (mm)	220 × 220	220 × 220
Voxel size (mm)	0.9 × 0.9x 1.0	0.9 × 0.9x 1.0
Matrix size	256 × 256	256 × 256
TR (ms)	2500	4.31
TE (ms)	4.3	1.81
Echo spacing (ms)	9.9	-
Inversion time (ms)	1100	-
Band width (Hx/pixel)	240	340
Average	1	2
Flip angle (°)	9	9
Acceleration method	GRAPPA	Compressed sensing
Number of acceleration factor	2	5
Number of slice	176	176
Scan time	5 min 48 s	2 min 44 s

*GRAPPA generalized auto calibrating partially parallel acquisitions, *TE* echo time, *TR* repetition time.

Gadolinium-based contrast agent (ProHance; Bracco, Milan, Italy) was injected intravenously at 0.2 ml/kg using a 3-way stopcock. The order of the post-contrast MR scanning was executed in random manner to minimize any potential bias due to the order of acquisition. Post-contrast MR scanning were executed just after the injection of contrast media. Fat suppression and pre-scan-based image intensity normalization were performed in both sequences. The total acquisition time was 5 min 48 s for conventional MPRAGE and 2 min 44 s for CS-VIBE^15^.

### Imaging analysis

All MRI scans were independently analyzed by two radiologists (10 years of experience in radiology) after a training session to familiarize themselves with the process of imaging analysis. The training session included 5 conventional MPRAGE and CS-VIBE scans which were excluded from the main analysis. Using the PACS system, each reader was blinded to the initial diagnosis, the sequence used, and the other observer’s results. Each reader assessed the images for each sequence separately with a 2-week interval to prevent recall bias. To evaluate diagnostic performance, we first determined whether any enhancing lesions were present followed by whether brain metastasis was present for both sequences.

Quantitative analyses were performed via a circular region of interest (ROI) measurements on axial images. For statistical analyses, the average values of both sides were considered the representative values. To increase the reliability of the extracted values, the rater measured the signal intensity (SI) three times, and the mean value was used for the analysis. The ROIs were located in the gray and white matter of the frontal lobe at the level of the centrum semiovale, central pons, and cerebellum, avoiding artifacts or enhancing lesions such as vessels. Using the data from these ROIs, the following quantitative metrics were acquired: (1) CNR for white and gray matter (CNR_WM/GM_); (2) SNR at the level of the centrum semiovale, pons, and cerebellum; (3) CNR and contrast ratio (CR) for enhancing lesions. The long diameters of enhancing lesions were measured and compared on both conventional MPRAGE and CS-VIBE. The CNR_WM/GM_ was calculated based on a previously published formula: (SI of white matter–SI of gray matter)/noise of white matter^16,17^. Due to the non-homogeneous noise distribution of the parallel acceleration images, we avoided direct measurement of the noise from the background and obtained the value from the standard deviation (SD) of the white matter instead, as described previously^16,18,19^.

For qualitative analysis, we assessed four categories: (1) overall image quality^20^; (2) motion artifact; (3) gray-white matter differentiation; (4) lesion conspicuity. Overall image quality and gray-white matter differentiation were scaled with following criteria: 1 = non-diagnostic image quality; 2 = severe blurring resulting in significant limitation in evaluation; 3 = moderate blurring that slightly compromised evaluation; 4 = slight blurring that did not compromise image assessment; and 5 = excellent image quality. Motion artifact was evaluated as follows: 1 = severe image artifacts; 2 = moderate artifacts; 3 = mild artifacts; 4 = minimal artifacts; 5 = no artifacts. For lesion conspicuity, we applied a 3-point scale as follows: 1 = a lesion whose borders were indistinguishable from the background; 2 = a lesion with blurry margins; 3 = sharp lesion margins. We also categorized the overall visual score into three groups (best, intermediate, and worst) and evaluated the preference between the two sequences.

### Statistical analysis

All data were analyzed using the software packages SPSS version 25.0 (SPSS Inc., Chicago, IL, USA) and MedCalc Statistical Software version 19.3.1 (MedCalc Software Ltd, Ostend, Belgium). We assessed the diagnostic accuracy (sensitivity, specificity, accuracy, positive predictive value, and negative predictive value) of conventional MPRAGE and CS-VIBE. We defined metastasis as biopsy- proven metastasis. If no pathology was available, lesions should satisfy all of the following conditions: (1) underlying cancer, (2) typical findings on MRI—uniform, punctate or ring-enhancement, T2/FLAIR hyperintensity with perilesional edema, (3) exclusion of other condition such as infection or active demyelinating disease. To evaluate diagnostic performance, we utilized final reference diagnosis made by a neuroradiologist with 13 years’ of experience in case of no pathology is available. The qualitative and quantitative imaging parameters were compared using the Wilcoxon signed-rank test. The correlation between conventional MPRAGE and CS-VIBE enhancing lesion detection and metastasis detection were assessed using Pearson correlation coefficient. The strength of agreement according to κ values was categorized as follows: poor, < 0.20; fair, 0.21–0.40; moderate, 0.41–0.60; good, 0.61–0.80; excellent, 0.81–1.00^21^. A *p* value < 0.05 was considered statistically significant.

## Results

Detailed clinical information about the study population is summarized in Table 1. Overall, CS-VIBE achieved comparable diagnostic performance to that of conventional MPRAGE in terms of sensitivity (conventional MPRAGE vs. CS-VIBE, 96.7% vs. 96.7%) and specificity (conventional MPRAGE vs. CS-VIBE, 99.6% vs. 100.0%). Diagnostic accuracy was also similar in both sequences (conventional MPRAGE vs. CS-VIBE, 99.1% vs. 99.4%) (Table 3).

**Table 3:** Diagnosis of brain lesions using conventional MPRAGE and CS-VIBE.

		Sensitivity (%)	Specificity (%)	Accuracy (%)	PPV (%)	NPV (%)
Enhancing brain lesion						
Overall	conventional MPRAGE	91.3	99.2	97.0	97.7	96.7
	CS-VIBE	93.5	99.6	97.9	98.9	97.5
Obs 1	conventional MPRAGE	95.7	99.2	98.2	97.8	98.3
	CS-VIBE	95.7	100.0	98.8	100.0	98.3
Obs 2	conventional MPRAGE	87.0	99.2	95.7	97.6	95.1
	CS-VIBE	91.3	99.2	97.0	97.7	96.7
Intracranial metastasis						
Overall	conventional MPRAGE	96.7	99.6	99.1	98.3	99.3
	CS-VIBE	96.7	100	99.4	100	99.3
Obs 1	conventional MPRAGE	100.0	99.3	99.4	96.8	100.0
	CS-VIBE	96.7	100.0	99.4	100.0	99.3
Obs 2	conventional MPRAGE	93.3	100.0	98.8	100.0	98.5
	CS-VIBE	96.7	100.0	99.4	100.0	99.3

**Figure 2:**
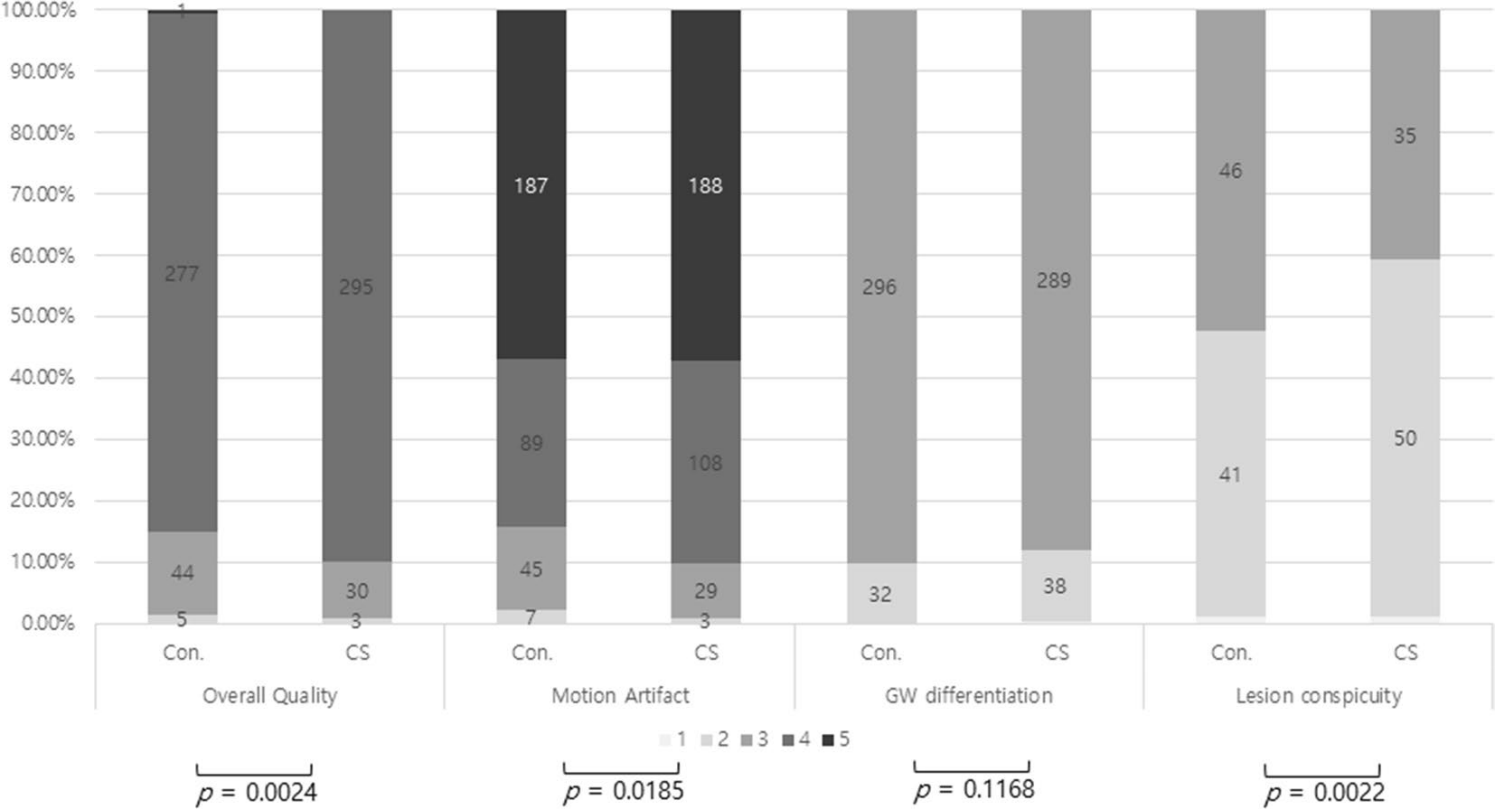
Image quality of conventional MPRAGE and CS-VIBE. Abbreviations: Con., conventional magnetization-prepared rapid-acquisition gradient-echo (MPRAGE); CS, compressed sensing volume interpolated breath-hold examination (CS-VIBE). Numbers of the bar plot indicate the grade of image quality as follows: (**a**) overall image quality and artifact (2 = poor, relevant limitations for image interpretation; 3 = adequate, moderate limitations for image interpretation; 4 = good, minimal limitations for image interpretation; 5 = excellent), (**b**) basal ganglia and sulcus demarcation, gray-white matter differentiation (1 = indistinguishable, 2 = blurry margins but distinguishable borders, 3 = adequate, 4 = good, 5 = excellent), and (**c**) enhancing lesion conspicuity (1 = a lesion whose borders were indistinguishable from background brain, 2 = a lesion with blurry margins, 3 = sharp lesion margins).

Qualitative analysis showed that the overall image quality of CS-VIBE was better than that of conventional MPRAGE (*p* = 0.002; Fig. 2, Table 4). In comparison with conventional MPRAGE, CS-VIBE also demonstrated better performance in terms of motion artifact reduction (*p* = 0.02; Fig. 2, Table 4). However, gray-white differentiation and enhancing lesion conspicuity were slightly better with conventional MPRAGE. A comparison of the overall visual score revealed that MPRAGE tended to provide the best image quality compared with that of CS-VIBE (32.9% vs. 28.4%, supplementary Fig. 1). When comparing the preference between the two sequences, readers indicated an equal preference in 75% (256 out of 328 observations) of cases, and conventional MPRAGE and CS-VIBE were preferred in 11.6% (38 out of 328 observations) and 13.4% (44 out of 328 observations) of cases, respectively (supplementary Fig. 1).

**Table 4:** Comparison of qualitative measurement.

	Conventional MPRAGE	CS-VIBE	*P* value
Overall quality	3.84 ± 0.42 (2–5)	3. 89 ± 0.34 (2–4)	0.002
Motion artifact	4.39 ± 0.80 (2–5)	4.47 ± 0.69 (2–5)	0.02
GW differentiation	2.90 ± 0.30 (2–3)	2.88 ± .0.34 (1–3)	0.12
Lesion conspicuity	2.50 ± 0.53 (1–3)	2.40 ± 0.52 (1–3)	0.002

*Numbers in parenthesis indicates range.

*GW* gray-white matter.

**Figure 3:**
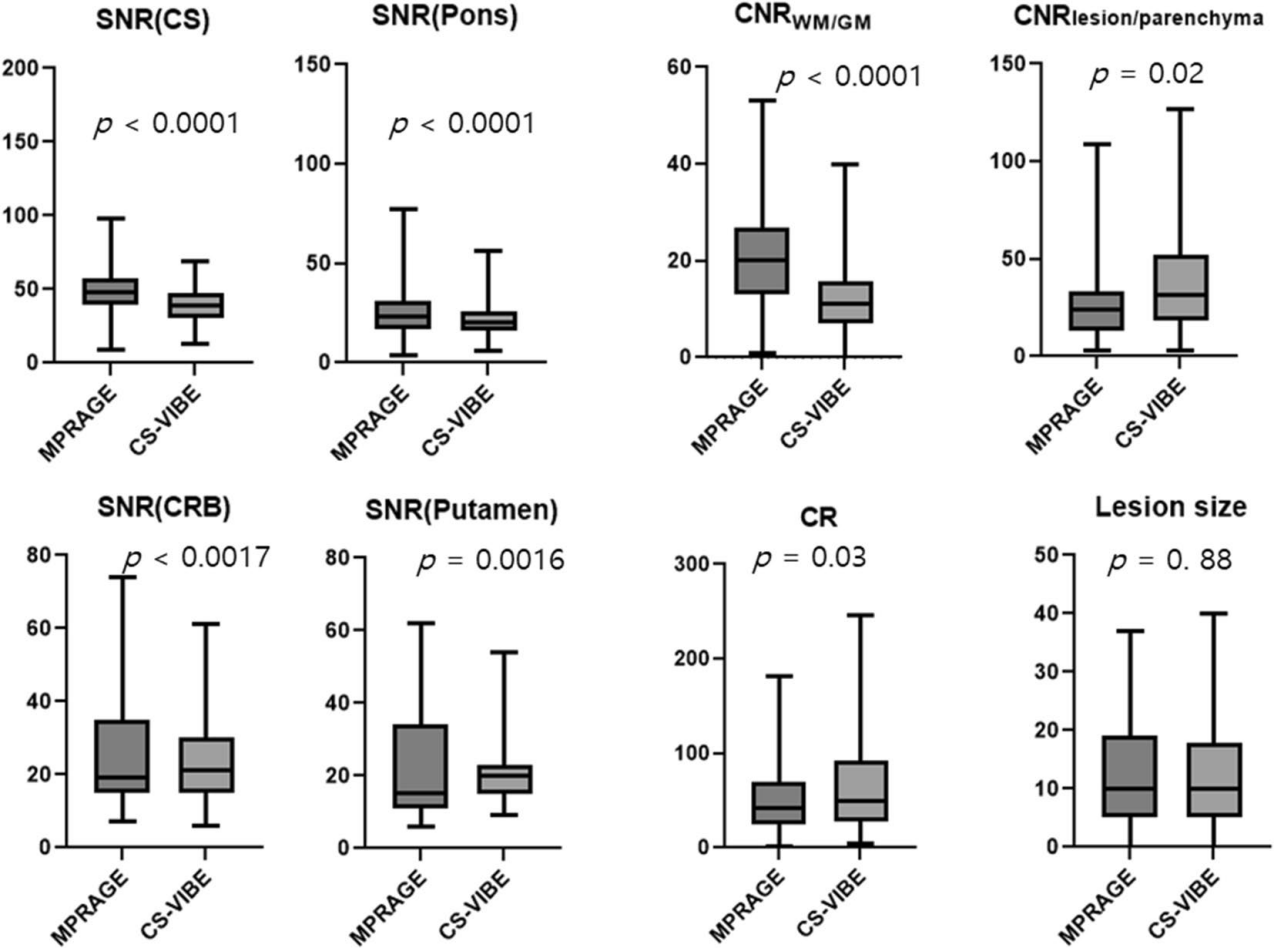
Quantitative image comparison between conventional MPRAGE and CS-VIBE. The SNR and CNR_WM/GM_ of CS-VIBE were lower than those of conventional MPRAGE in the centrum semiovale, pons, and cerebellum. The CR of CS-VIBE was higher than that of MPRAGE. The CNR_lesion/parenchyma_ and contrast rate of CS-VIBE were higher than those of conventional MPRAGE. Size measurement of enhancing lesions were similar in two sequences. Abbreviations: CS, centrum semiovale; CRB, cerebellum.

Quantitative imaging parameter analysis revealed the lower SNR and CNR_WM/GM_ of CS-VIBE compared with those of conventional MPRAGE (*p* < 0.05; Fig. 3, Supplementary Table 1). For enhancing lesions, CS-VIBE demonstrated higher CNR _lesion/parenchyma_ (Fig. 3, conventional MPRAGE vs. CS-VIBE, 27.59 ± 19.91 vs. 37.58 ± 28.83,* p* = 0.02) and CR (Fig. 3, conventional MPRAGE vs. CS-VIBE, 53.23 ± 39.39 vs. 64.05 ± 50.62,* p* = 0.03). The lesion size was similar between both sequences (Fig. 3, conventional MPRAGE vs. CS-VIBE, 14.36 ± 13.66 mm vs. 14.06 ± 13.75 mm,* p* = 0.88). The ICCs between the two sequences in detecting enhancing lesions and metastasis were excellent (κ = 0.98 and 0.97 respectively).

## Discussion

**Figure 4:**
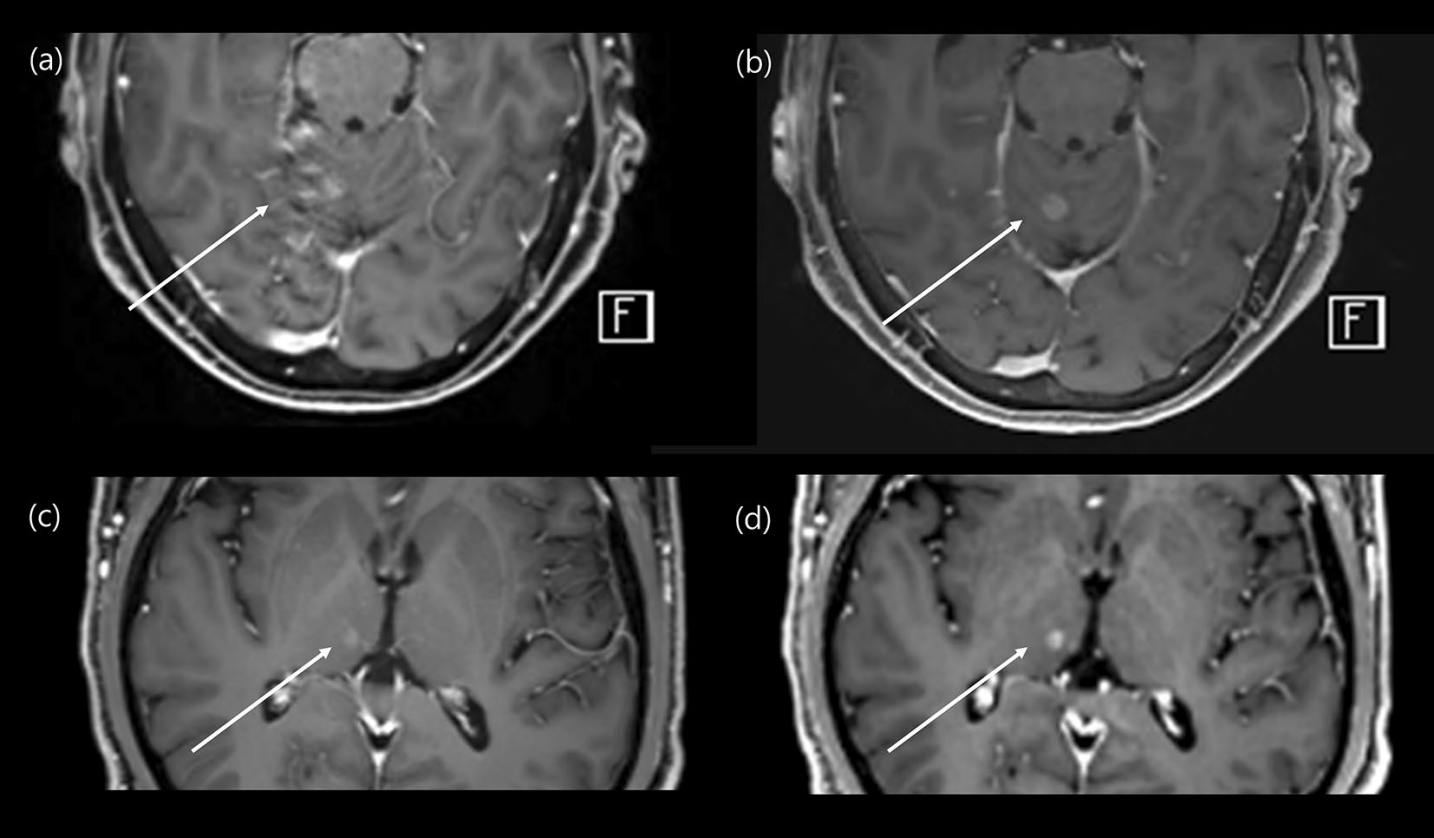
Comparison of an enhancing intracranial lesion on conventional MPRAGE and CS-VIBE. A 67-year-old female and a 59-year-old male with intracranial metastasis from lung cancer in the right cerebellum (**a**, **b**) and right thalamus (**c**, **d**). Conventional MPRAGE (**a**, **c**) showed an irregular and indistinct enhancement whereas CS-VIBE (**b**, **d**) showed a discrete, nodular enhancement, which indicated metastasis.

In this study, we demonstrated similar diagnostic accuracy of contrast-enhanced CS-VIBE and conventional post-contrast MPRAGE for the evaluation of intracranial metastasis. Using CS-VIBE, we could acquire a high resolution intracranial post-contrast image covering the whole brain in less than 3 min, which is less than half of the scan time of conventional MPRAGE. In addition, CS-VIBE exhibited better performance in terms of the enhancement degree and CNR_lesion/parenchyma_. Experienced radiologists rated CS-VIBE as providing a better overall image quality with less motion artifact; however, there was no significant difference in the preference between CS-VIBE and conventional post-contrast MPRAGE (Fig. 4). These findings support the broader application of a fast scan protocol using CS-VIBE sequences in the evaluation of patients with possible intracranial metastasis.

**Figure 5:**
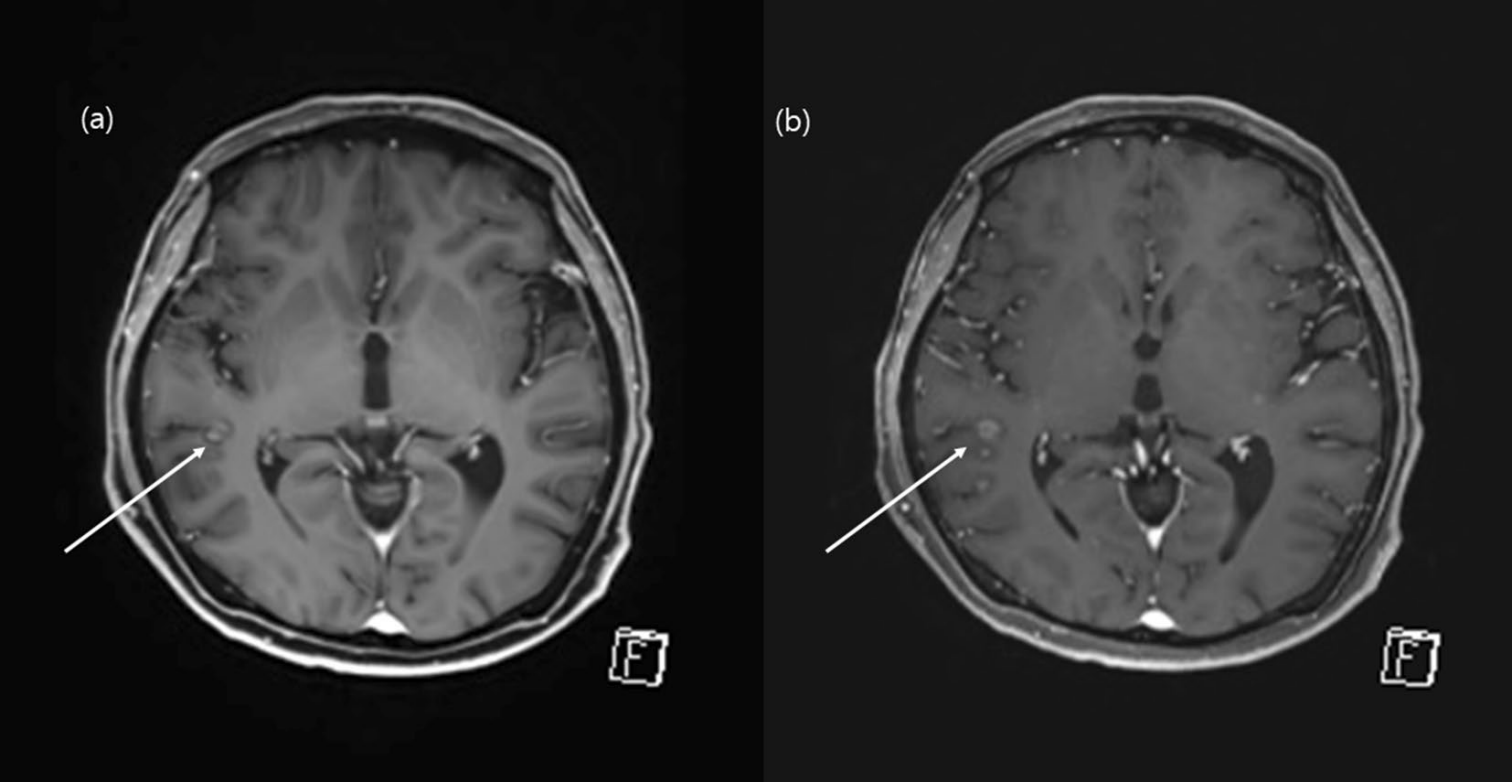
Comparison of an enhancing intracranial lesion on conventional MPRAGE and CS-VIBE. A 52-year-old female with intracranial metastasis from lung cancer. A tiny nodular rim-enhancement in the right temporal cortex indicated intracranial metastasis. Both conventional MPRAGE (**a**) and CS-VIBE (**b**) showed a similar shape and enhancement; however basal ganglia structures and white gray matter differentiation were clearer with conventional MPRAGE.

**Figure 6:**
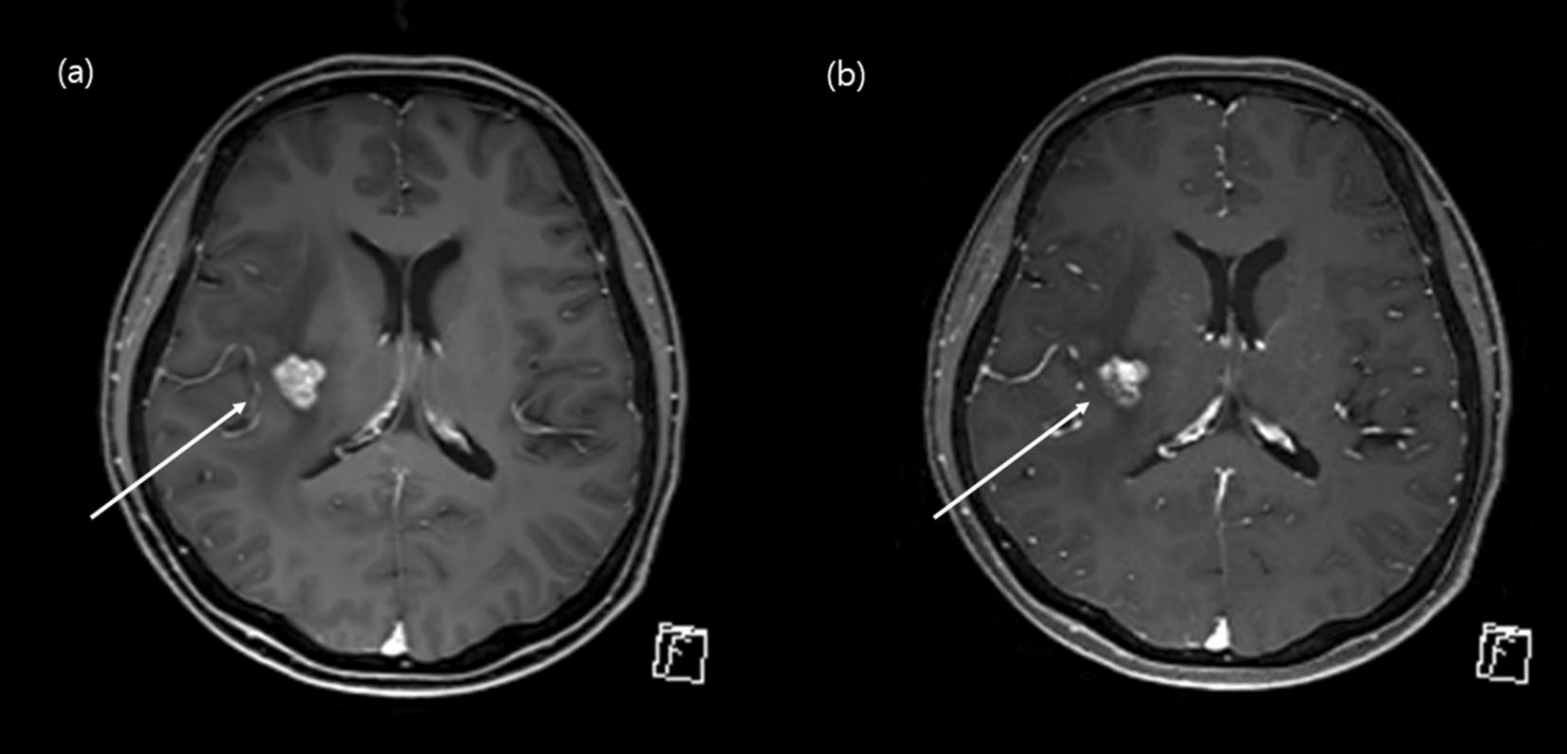
Comparison of an enhancing intracranial lesion on conventional MPRAGE and CS-VIBE. A 52-year-old female with intracranial metastasis from breast cancer. A small lobulated contour enhancement in the right basal ganglia indicated intracranial metastasis. Both conventional MPRAGE (**a**) and CS-VIBE (**b**) demonstrate similar contour and enhancement.

We demonstrated that the contrast-enhanced CS-VIBE may be a feasible and reliable accelerated MR method for the detection of enhancing intracranial lesions (Figs. 5 and 6). Both MPRAGE and VIBE are known to provide T1-weighted images and use spoiled GRE sequences with ultrashort TRs^8^. MPRAGE requires a prolonged scan time due to the extended magnetization preparation and recovery time before rapid GRE acquisition. On the other hand, VIBE utilizes a fast low-angle shot approach to produce T1-weighted image contrast in steady state imaging. In addition, the VIBE is superior in contrast-enhancement as the short TR of VIBE could increase the SNR and CNR of enhancing lesions^8,9^. Previous studies found that contrast-enhanced VIBE sequence was an effective, alternative approach to MPRAGE imaging for 3D T1-weighted imaging of the brain which demonstrated superior lesion detection and lesion conspicuity^8,9^. In this study, we applied CS to further accelerate brain imaging. CS has been used in clinical settings to combine incoherent k-space sampling during acquisition with a dedicated iterative reconstruction algorithm^22^. Although several studies have shown that the CS technique is useful for reducing the scan time when evaluating the vessel walls or neurovascular compression syndrome^18,23,24^, CS-VIBE has not been used in various sequences other than the dynamic imaging of the liver or breast with a high spatio-temporal resolution^10,11,25^. Although we previously found that accelerated CS-VIBE MRI could be a reliable method for the diagnosis of facial neuritis with half the scan time of conventional MPRAGE, the application of CS-VIBE to the CNS remains limited^15^.

In this study, the SNR of conventional MPRAGE was higher than that of CS-VIBE; however, the CNR _lesion/parenchyma_ was higher for CS-VIBE. Although a direct comparison of the SNR and CNR between conventional MPRAGE and CS-VIBE may be inappropriate as the noise distribution of the sequences is inhomogeneous as a result of (1) geometrical factors caused by parallel acceleration technique or regularization and (2) de-noising of both techniques^26–30^, SNR and CNR still play useful role in comparing the performance of the two sequences in case of related qualitative image parameters are preserved^26,29^. To complement quantitative analysis, we also conducted qualitative analysis to determine the possibility of actual clinical application. CS-VIBE was slightly better in terms of the overall image quality and motion artifact reduction; however conventional MPRAGE was advantageous for gray-white matter differentiation and lesion conspicuity.

Several studies have reported using a fast scan protocol with various accelerated 3D acquisition techniques, which showed lower image quality compared with that of a standard contrast-enhanced 3D T1-weighted sequence^31–33^. For example, a recent study using a contrast-enhanced 3D- fast low-angle shot (FLASH) sequence showed reduced susceptibility and motion artifacts with the decreased conspicuity of small lesions due to a lower SNR when compared with conventional MPRAGE^32^. In addition, another recent study using ultrafast 3D-EPI technique showed lower image quality and more susceptibility artifacts even though the technique could reduce the scan time^31^.

Although we obtained acceptable results without considerable degradation in image quality when diagnosing intracranial metastasis using CS-VIBE, one of the well-known drawback of the CS technique is image blurring especially at the white–gray matter border, which can be caused by a high acceleration factor or random under-sampling of the k-space at the peripheral location, as well as iterative reconstruction^26,29,30^. Due to the image blurring at the white–gray matter border, CS may have difficulty in detecting subtle changes in the cortico-subcortical areas^26^. Therefore, despite the acceptable results obtained from diagnosing intracranial metastasis, CS-VIBE may not be clinically suitable for diagnosing small peripheral lesions. In addition, compared with conventional sequences, the use of CS may be restricted as it usually requires more computational power^20,34^. Recently, CS-MP2RAGE was introduced and tested in evaluating brain volume as an alternative of standard MPRAGE showing same quality and higher contrast than the standard MPRAGE with almost half of the scan time^35,36^. We are expecting to apply CS-MP2RAGE and validate the sequence in diagnosing intracranial lesions with future studies.

There are several limitations in this study. First, selection bias could exist because this study was performed at a single referral center with relatively small number of patients. Further study with larger sample size would be beneficial for the more accurate validation of the sequence. Second, although the raters were blinded to the pulse sequence during qualitative evaluation, they might recognize the imaging features of each sequence, which could cause observer bias. Moreover, image quality could be affected by the acquisition order considering that images acquired later during examination could have more motion artifacts. We tried to minimize bias (1) by randomizing the order of acquisition during the study, (2) reducing scan duration using parallel imaging, and (3) undergoing quantitative analyses along with the qualitative evaluation. Also, it would be helpful if we additionally check whether the readers correctly identify each sequence.

Third, the readers who attended the study are all experienced neuroradiologists which could affect the result of diagnostic performance. It would be beneficial to involve readers who are in various stage of experience in neuro imaging to investigate the actual clinical feasibility.

Fourth, not all intracranial metastases were confirmed pathologically. As small lesions could not be diagnosed by surgical excision and biopsy, we referred to the clinical diagnosis in case no pathology was available. Finally, we only compared clinical feasibility between the conventional MPRAGE and CS-VIBE, so it would be better to compare other fast scans to find out the best suitable fast scan in diagnosing intracranial metastasis in future studies.

In conclusion, compared with conventional MPRAGE, CS-VIBE demonstrated comparable diagnostic performance with acceptable image quality and a shorter scan time in the diagnosis of intracranial metastasis in cancer patients. CS-VIBE exhibited similar or better performance in detecting enhancing lesions in terms of the enhancement degree and CNR _lesion/parenchyma_. Given the shorter scan time and comparable diagnostic performance, CS-VIBE could be an alternative accelerated post-contrast MRI method to conventional MRPAGE in daily practice.

## Supplementary Information

Supplementary Information 1.

Supplementary Information 2.

## Data Availability

The datasets used and/or analysed during the current study available from the corresponding author on reasonable request, but there could be certain process to release the data due to the local policy.

## Abbreviations

CS-VIBE: Compressed sensing volume-interpolated breath-hold examination
MPRAGE: Magnetization-prepared rapid-acquisition gradient-echo
CNR: Contrast-to-noise ratio
SNR: Signal-to-noise ratio
