# Supplementary material for: Clinical feasibility of CS-VIBE accelerates MRI techniques in diagnosing intracranial metastasis
Source: Sci Rep. 2023 Jun 20;13:10012. doi: 10.1038/s41598-023-37148-3 (PMC10282025; doi:10.1038/s41598-023-37148-3)
Supplement: Supplementary file 1 — Supplementary Information 1. [file 41598_2023_37148_MOESM1_ESM.docx]

**Supplementary table 1. Comparison of Quantitative Analysis Scores of conventional MPRAGE and CS-VIBE.**

|  | **Conventional MPRAGE** | **CS-VIBE** | **P-value** |
| --- | --- | --- | --- |
| SNR (CS) | 49.82± 16.80 | 38.63 ± 12.75 | <0.0001 |
| SNR (Pons) | 25.23± 12.63 | 21.46 ± 8.61 | <0.0001 |
| SNR (CRB) | 25.82± 15.96 | 23.43 ± 10.67 | 0.0017 |
| SNR (Putamen) | 22.64 ± 14.20 | 20.21 ± 7.06 | 0.0016 |
| CNR _white/gray matter_ | 20.04 ± 10.57 | 11.42 ± 6.20 | <0.0001 |
| CNR _lesion/parenchyma_ | 27.59 ± 19.91 | 37.58 ± 28.83 | 0.02 |
| CR | 53.23 ± 39.39 | 64.05 ± 50.62 | 0.03 |
| Enhancing lesion size^*^ | 14.36 ± 13.66 | 14.06 ± 13.75 | 0.88 |

*size was measured mm
