# Supplementary figures and images for: Clinical feasibility of CS-VIBE accelerates MRI techniques in diagnosing intracranial metastasis
Source: Sci Rep. 2023 Jun 20;13:10012. doi: 10.1038/s41598-023-37148-3 (PMC10282025; doi:10.1038/s41598-023-37148-3)

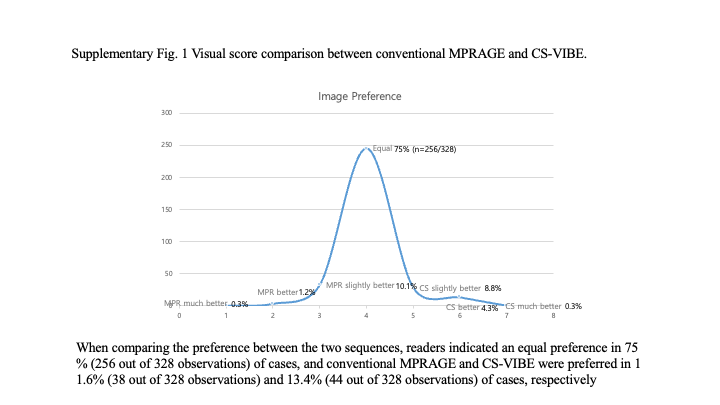

Supplement: Supplementary file 2 — Supplementary Information 2. [file 41598_2023_37148_MOESM2_ESM.tiff]
